# Predictors of Inappropriate Use of Diagnostic Tests and Management of Bronchiolitis

**DOI:** 10.1155/2017/9730696

**Published:** 2017-07-03

**Authors:** Lorena Sarmiento, Gladys E. Rojas-Soto, Carlos E. Rodríguez-Martínez

**Affiliations:** ^1^Department of Pediatrics, Clinica Universitaria Colombia, Fundacion Universitaria Sanitas, Bogota, Colombia; ^2^Department of Pediatric Respiratory Medicine, Hospital El Pino, University of Santiago de Chile (USACH), Santiago, Chile; ^3^Department of Pediatrics, School of Medicine, Universidad Nacional de Colombia, Bogota, Colombia; ^4^Department of Pediatric Pulmonology and Pediatric Critical Care Medicine, School of Medicine, Universidad El Bosque, Bogota, Colombia

## Abstract

**Background:**

The aim of the present study was to determine predictors of inappropriate use of diagnostic tests and management of bronchiolitis in a population of hospitalized infants.

**Methods:**

In an analytical cross-sectional study, we determined independent predictors of the inappropriate use of diagnostic tests and management of bronchiolitis in a population of hospitalized infants. We defined a composite outcome score as the main outcome variable.

**Results:**

Of the 303 included patients, 216 (71.3%) experienced an inappropriate use of diagnostic tests and treatment of bronchiolitis. After controlling for potential confounders, it was found that atopic dermatitis (OR 5.30; CI 95% 1.14–24.79; *p* = 0.034), length of hospital stay (OR 1.48; CI 95% 1.08–2.03; *p* = 0.015), and the number of siblings (OR 1.92; CI 95% 1.13–3.26; *p* = 0.015) were independent predictors of an inappropriate use of diagnostic tests and treatment of the disease.

**Conclusions:**

Inappropriate use of diagnostic tests and treatment of bronchiolitis was a highly prevalent outcome in our population of study. Participants with atopic dermatitis, a longer hospital stay, and a greater number of siblings were at increased risk for inappropriate use of diagnostic tests and management of the disease.

## 1. Introduction

Acute bronchiolitis represents the most important cause of lower respiratory tract infection during the first year of life and is the leading reason for hospitalization of infants beyond the neonatal period [1]. The disease is usually associated with substantial direct and indirect costs, not only for healthcare systems but also for families and society as a whole [2]. Although bronchiolitis is an extremely common disease and several clinical practice guidelines (CPGs) of acceptable quality have been developed [3], there is still a significant use and overuse of medications with insufficient evidence of effectiveness [4] and use of unnecessary diagnostic tests [5], generating unnecessary and costly resource use with no improvement in important clinical outcomes [6]. It is important to identify predictors of inappropriate use of diagnostic tests and management of bronchiolitis, which would help to plan more focused strategies and more targeted efforts in hope of achieving better disease management. An implementation strategy of CPGs that deal with the identified predictors could help to reduce the inappropriate use and overuse of diagnostic tests and medications that lack strong evidence that supports a recommendation for their routine use [4]. This implementation strategy could lead to the economical and effective use of health care services and the avoidance of wasting resources without reducing the quality of these services, finally contributing to the improvement of disease outcomes. To date, only a few studies have identified predictors of inappropriate use of diagnostic tests and management of bronchiolitis, showing that the region or the community where the patients were treated [7, 8] and whether patients were treated in a non-university-affiliated hospital were independent predictors of inappropriate management of bronchiolitis [7]. However, there is a need for further studies, which ideally should account for patient-related variables such as disease severity and the presence of comorbidities and atopic conditions, not only in patients but also in their families. Accordingly, the aim of the present study was to identify potential factors associated with the inappropriate use of diagnostic tests and management of bronchiolitis in a population of infants hospitalized for acute viral bronchiolitis in the Fundación Hospital La Misericordia, a university-affiliated hospital located in the metropolitan area of Bogota, Colombia.

## 2. Material and Methods

### 2.1. Bronchiolitis Clinical Practice Guideline (CPG)

At the time of the study's inception, an evidence-based bronchiolitis institutional CPG was available at the Fundación Hospital La Misericordia and served as a basis for guidance about which diagnostic tests and medications should be used in infants treated in the hospital for any acute viral bronchiolitis episode [9].

The bronchiolitis CPG recommends against routine use of diagnostic tests such as a hemogram, C-reactive protein, and procalcitonin and suggests that C-reactive protein and procalcitonin should be ordered only for infants with suspected serious bacterial infections. Likewise, the CPG recommends against the routine use of chest X-rays, suggesting that they should be ordered only for infants with diagnostic uncertainty, severe disease, or an atypical disease course. With respect to pharmacologic therapy, the CPG also recommends against the routine use of inhaled bronchodilators (beta 2 agonists, anticholinergics), nebulized epinephrine, and anti-inflammatories (inhaled or systemic corticosteroids) but allows for the option of a monitored trial of inhaled bronchodilators or nebulized epinephrine, continuing their administration only if there is a documented positive clinical response to the trial using an objective means of evaluation. Finally, the CPG also recommends that nebulized hypertonic saline be administered to infants hospitalized for bronchiolitis in order to shorten hospital stay but recommends against its use in the emergency department.

### 2.2. Study Design and Procedures

An analytical cross-sectional study was performed during the period from March to June 2014, in a random sample of patients aged <2 years hospitalized in the Fundacion Hospital de La Misericordia with a diagnosis of acute bronchiolitis (ICD-10 codes J21, J21.0, J21.1, J21.8, and J21.9). We chose this time period because it roughly coincides with the main bronchiolitis season in the city [10]. After reviewing the electronic medical records (EMRs), we collected the following demographic and clinical information: date of admission, age, gender, occurrence of breastfeeding for at least six months, nursery attendance, number of siblings, number of days with respiratory symptoms, presence of underlying disease conditions, previous use of oxygen therapy, personal history or diagnosis of atopic dermatitis, and parental history of asthma. Likewise, we collected information related to the use of diagnostic tests and use of pharmacologic therapy when diagnosing and/or treating bronchiolitis. Finally, we collected information related to the care or the disease-severity parameters, such as length of hospital stay, need for home oxygen administration, need for pediatric intensive care unit (PICU) admission, need for endotracheal intubation, and mortality.

The study protocol was approved by the local ethics board.

### 2.3. Outcome Definition

As the primary outcome of interest, we defined a priori a composite outcome score, which we termed “inappropriate use of diagnostic tests and treatment of bronchiolitis.” This composite outcome score aggregated the use of diagnostic tests (hemogram, C-reactive protein, procalcitonin, chest radiography, and tests for detection of respiratory viruses) and the use of pharmacologic therapy (inhaled or nebulized beta 2 agonists, inhaled or nebulized anticholinergics, nebulized epinephrine, inhaled corticosteroids, systemic corticosteroids, antibiotics, and nebulized hypertonic saline). Each of these 12 items had a binary score: 0 indicating that the diagnostic test or the medication was not used and 1 indicating that they were used (except for the use of nebulized hypertonic saline, which also had a binary score, but with 0 indicating that the medication was used and 1 indicating that it was not used). The use of C-reactive protein and procalcitonin was deemed as appropriate in patients with suspected or confirmed serious bacterial infections, the use of chest X-rays was deemed as appropriate in patients with severe disease, or with atypical disease course, and the use of inhaled beta 2 agonists and epinephrine was deemed as appropriate only if they were continued after a positive clinical response to a monitored trial. The total score was calculated by adding up the scores for the 12 items, resulting in a total score ranging from 0 to 12 (best to worst). A composite outcome score of >3 was designated as “inappropriate use of diagnostic tests and treatment of bronchiolitis.” The sole use of anticholinergics (nebulized or inhaled) or corticosteroids (inhaled or systemic) was deemed to be an “inappropriate use of diagnostic tests and treatment of bronchiolitis,” independent of the composite outcome score. A sensitivity analysis was performed using a range of cutoff values from 1 to 5 of the composite outcome score for defining “inappropriate use of diagnostic tests and treatment of bronchiolitis.”

### 2.4. Statistical Analysis

Continuous variables are presented as mean ± standard deviation (SD) or median (interquartile range [IQR]), whichever is appropriate. Categorical variables are presented as numbers (percentage). Differences between continuous variables were analyzed using the unpaired *t*-test or Wilcoxon's signed rank test, whichever was appropriate. Associations between categorical variables and the outcome variable were analyzed using the chi-square test or Fisher's exact test, whichever was appropriate. To identify factors independently associated with an inappropriate use of diagnostic tests and treatment of bronchiolitis, we used logistic regression models, adjusting for potential confounding variables. The predictive variables included in the models were the age of the patients, the number of days with respiratory symptoms, a parental history of asthma, and the presence of at least one underlying disease or condition associated with severe disease. All statistical tests were two-tailed, and the significance level used was *p* < 0.05. The data were analyzed with Statistical Package Stata 12.0 (Stata Corporation, College Station, TX).

## 3. Results

### 3.1. Study Population

A total of 303 infants with a diagnosis of acute bronchiolitis were evaluated. Of the 303 included patients, 176 (58.1%) were males, and the median IQR age was 3.0 (1.0–7.0) months. The age group distribution was 203 (67.0%) less than 6 months, 92 (30.4%) between 6 and 12 months, and the remaining 8 (2.6%) between 13 and 24 months. Regarding the presence of a history of personal or familiar atopic diseases, 45 (14.9%) patients had a history of parental asthma and 13 (4.3%) a previous diagnosis of atopic dermatitis. All the related demographic and clinical variables, according to the appropriateness of bronchiolitis use of diagnostic tests and treatment, are presented in Table 1.

### 3.2. Use of Diagnostic Tests

The use of diagnostic tests in the study period was as follows: hemogram in 159 (52.5%) patients, C-reactive protein in 52 (17.2%), procalcitonin in 6 (2.0%), chest X-ray in 209 (69.0%), and tests for detection of respiratory viruses in 207 (68.3%).

### 3.3. Pharmacologic Therapy

Rates of use of medications were as follows: inhaled or nebulized beta 2 agonists in 271 (89.4%) patients, inhaled or nebulized anticholinergics in 30 (9.9%), nebulized epinephrine in 94 (31.0%), inhaled corticosteroids in 53 (17.5%), systemic corticosteroids in 52 (17.2%), antibiotics in 89 (29.4%), and nebulized hypertonic saline in 48 (15.8%).

### 3.4. Care or Bronchiolitis-Severity Parameters

With respect to the care or the disease-severity parameters, the median (IQR) of the length of hospital stay was 4.0 (3.0–5.0). Out of the total of 303 patients included in the study, 98 (32.3%) had a continued need for oxygen therapy at home, 4 (1.3%) required admission to the PICU, 1 (0.3%) required endotracheal intubation, and none died due to the respiratory disease.

### 3.5. Inappropriate Use of Diagnostic Tests and Treatment of Bronchiolitis

Based on the above-mentioned definition, it was considered that 216 (71.3%) patients experienced an inappropriate use of diagnostic tests and treatment of bronchiolitis. This was mainly due to the use of beta 2 agonists in 271 (89.4%) patients, a lack of the use of nebulized hypertonic saline in 252 (83.2%), the use of diagnostic tests such as chest X-rays in 209 (69.0%), and a hemogram in 159 (52.5%). When compared with patients with an appropriate use of diagnostic tests and treatment of the disease, patients with an inappropriate use of diagnostic tests and treatment had a significantly longer hospital stay (4.0 [3.0–6.0] versus 3.0 [2.0–4.0], *p* < 0.001) and a significant higher isolation rate of respiratory syncytial virus (RSV) (44.0% versus 23.0%, *p* < 0.001). The remaining demographic and clinical characteristics that were measured did not differ between the two groups of patients (Table 1).

### 3.6. Predictors of Inappropriate Use of Diagnostic Tests and Treatment of Bronchiolitis through Multivariate Analysis

Multivariate analyses were conducted to determine independent predictors of the outcome variable. The predictor variables included in the multivariate models were age, number of brothers and sisters, number of days with respiratory symptoms, presence of at least one underlying disease condition, personal history or diagnosis of atopic dermatitis, parental history of asthma, previous use of oxygen therapy, RSV isolation, and length of hospital stay. After controlling for these potential confounders, it was found that a personal history or diagnosis of atopic dermatitis (OR 5.30; CI 95% 1.14–24.79; *p* = 0.034), length of hospital stay (OR 1.48; CI 95% 1.08–2.03; *p* = 0.015), and the number of siblings (OR 1.92; CI 95% 1.13–3.26; *p* = 0.015) were independent predictors of an inappropriate use of diagnostic tests and treatment of bronchiolitis in our sample of patients (Table 2). In the sensitivity analysis using cutoff values from 1 to 5 of the composite outcome score, predictors of “inappropriate use of diagnostic tests and treatment of bronchiolitis” did not change substantially (data not shown).

## 4. Discussion

The results of this study show that there was a high prevalence of an inappropriate use of diagnostic tests and treatment of bronchiolitis in infants hospitalized in a third-level teaching referral hospital. Participants with a personal history or diagnosis of atopic dermatitis, a longer hospital stay, and a greater number of siblings were at increased risk for inappropriate use of diagnostic tests and treatment.

We are confident that our results may improve the knowledge about patient and disease characteristics, which can serve as a basis for planning more targeted implementation strategies that could help to improve the rational use of diagnostic tests and medications that lack strong evidence that supports a recommendation for their routine use in bronchiolitis. Although we identified a set of predictors independently associated with an inappropriate diagnosis and management of bronchiolitis not previously reported in the literature, our results are in line with previous studies that have also identified disease accompanying conditions or disease characteristics themselves as predictors of the appropriateness of the treatment. Henao-Villada et al. identified the presence of an underlying disease condition and bronchiolitis caused by viruses other than respiratory syncytial virus (RSV) as independent predictors of an inappropriate diagnosis and treatment of bronchiolitis in a population of children admitted to the emergency department or hospitalized for the disease. Additionally, age was inversely correlated with the appropriateness of the treatment, suggesting that younger patients have a greater probability of being adequately diagnosed and treated [11]. In our study, we identified that a personal history or diagnosis of atopic dermatitis and a longer hospital stay also independently predicted an inappropriate diagnosis and management of the disease. Taken together, these findings would seem to suggest that the less typical or less pure form the disease has, the lower the probability of having an appropriate diagnosis and management. The typical patient with acute viral bronchiolitis is a small infant with a demonstrated infection by RSV, which usually requires a short-term stay when hospitalization is necessary [12]. For this reason, it is plausible to consider that there is a greater probability of requesting diagnostic tests or prescribing the use of medications not routinely recommended in CPGs when clinicians deal with patients with bronchiolitis without this typical clinical presentation. For example, clinicians could consider that an infant suffering from bronchiolitis with a personal history or diagnosis of atopic dermatitis has a high probability of future asthma symptoms and could be more prone to treat the bronchiolitis episode in the same manner as an asthma exacerbation episode. Likewise, clinicians treating an older infant with initial symptoms suggestive of bronchiolitis but with prolonged symptom duration may feel compelled to use diagnostic tests or to prescribe medications that are not routinely recommended in evidence-based CPGs, probably in order to consider the possibility of an alternative diagnosis, or even because of the pressure of parents who feel that their child is not getting better. Other studies have identified other factors unrelated to the disease or the patient himself as predictors of inappropriate management of bronchiolitis, such as the region, the community, and the type of university affiliation of the hospital where the infants were treated [7, 8]. Nevertheless, these results are difficult to compare with ours, because we performed a single-center study. We found that a greater number of siblings was associated with an increased risk of inappropriate diagnosis and management of bronchiolitis; however the reason for this association is not entirely clear. A reasonable explanation for this association may be due to the fact that the number of siblings has been reported as an independent predictor for recurrent wheezing following acute bronchiolitis [13]. It is probable that some of these recurrent wheezing episodes have been diagnosed as acute viral bronchiolitis but have been treated as acute asthma exacerbations.

A number of potential limitations need to be considered. First, we used a nonvalidated definition of inappropriate diagnosis and treatment of bronchiolitis. Although we based this definition on the recommendations provided by a current evidence-based CPG, there is a gap in the literature for evaluating the actual appropriateness of bronchiolitis management for specific indications. For example, it has been reported that atopic dermatitis aggravates the allergic airways inflammation in patients with acute viral bronchiolitis [14]. Therefore, it is probable that treating patients with acute viral bronchiolitis and coexisting atopic dermatitis with corticosteroids could not be considered completely inappropriate. However, current available evidence does not support such a practice. Second, since this study was based on an EMR review, assessing the appropriateness of the diagnosis and management of bronchiolitis was based on information documented in the patient's EMR. It is possible that a patient may have had additional symptoms or findings that would have altered the appropriateness of the diagnosis and management of the disease. Third, patients diagnosed and treated in other settings (emergency department, outpatients) were not included in the present study, thus limiting its generalizability. Finally, as opposed to other studies in which the variability and the appropriateness of bronchiolitis management were determined in a representative sample of all Health Care Centers and all regions of the country [8], our study was limited to patients hospitalized at a single pediatric hospital. Therefore, our results need to be interpreted with caution because they could not be generalizable to all bronchiolitis patients. However, we are confident that our results represent an excellent initial step toward designing interventions that include a more rational approach to the diagnosis and treatment of patients with bronchiolitis.

## 5. Conclusions

In conclusion, the findings of the present study show that there was a high prevalence of the inappropriate use and overuse of medications that have insufficient evidence of effectiveness and the use of unnecessary diagnostic tests in a population of hospitalized infants with acute viral bronchiolitis. Patients with a personal history or diagnosis of atopic dermatitis, a longer hospital stay, and a greater number of siblings were at increased risk of inappropriate diagnosis and treatment. Future research on a validated definition of an inappropriate diagnosis and treatment of bronchiolitis should be undertaken.

## Figures and Tables

**Table 1:** Demographic characteristics and clinical information of the patients included in the study, according to the appropriateness of bronchiolitis diagnosis and treatment.

Variable	Patients with inadequate diagnosis and treatment^*∗*^ (*n* = 216)	Patients with adequate diagnosis and treatment (*n* = 87)	*p* value
Age (months; median (interquartile range–IQR))	3.0 (1.0–7.0)	3.0 (1.0–6.0)	0.966
Gender, M/F	131/85	45/42	0.154
Presence of breastfeeding for at least six months	31 (14.4%)	14 (16.1%)	0.665
Nursery attendance	20 (9.3%)	7 (8.0%)	0.780
Number of siblings	1.0 (0.0–1.0)	0.0 (0.0–1.0)	0.306
Number of days with respiratory symptoms			
Less than 24 hours	19 (8.8%)	12 (13.8%)	0.211
Between 24 and 72 hours	192 (88.8%)	71 (81.6%)	0.095
More than 72 hours	3 (1.4%)	1 (1.1%)	1.00
Presence of underlying disease conditions	26 (12.0%)	13 (14.9%)	0.513
Previous use of oxygen therapy	6.0 (6.9%)	5.0 (2.3%)	0.055
Personal history or diagnosis of atopic dermatitis	7.0 (8.0%)	6.0 (2.8%)	0.073
Parental history of asthma	32 (14.8%)	13 (14.9%)	0.846
Respiratory syncytial virus isolation	95 (44.0%)	20 (23.0%)	**<0.001**
Length of hospital stay	4.0 (3.0–6.0)	3.0 (2.0–4.0)	**<0.001**
Need for home oxygen administration	76 (35.2%)	22 (25.3%)	0.082
Need for pediatric intensive care unit	4 (1.9%)	0 (0.0%)	0.675
Need for endotracheal intubation	1 (0.5%)	0 (0.0%)	1.00
Mortality	0 (0.0%)	0 (0.0%)	0.496

^*∗*^Significant (*p* < 0.05) associations.

**Table 2:** Predictors of inappropriate diagnosis and treatment of bronchiolitis through multivariate analysis.

Variable	Odds ratio (OR) (95% CI)	*p* value
Age	0.95 (0.84–1.07)	0.378
Number of siblings	1.92 (1.13–3.26)	**0.015**
Number of days with respiratory symptoms		
Less than 24 hours	1.00	—
Between 24 and 72 hours	1.19 (0.23–6.24)	0.834
More than 72 hours	2.93 (0.68–12.69)	0.150
Presence of at least one underlying disease condition	4.02 (0.96–16.90)	0.057
Previous use of oxygen therapy	2.34 (1.78–30.74)	0.518
Personal history or diagnosis of atopic dermatitis	5.30 (1.14–24.79)	**0.034**
Parental history of asthma	1.46 (0.52–4.12)	0.470
Length of hospital stay	1.48 (1.08–2.03)	**0.015**
